# How much can the orientation of G's eigenvectors tell us about genetic constraints?

**DOI:** 10.1002/ece3.306

**Published:** 2012-07-04

**Authors:** Daniel Berner

**Affiliations:** Zoological Institute, University of BaselVesalgasse 1, CH-4051, Basel, Switzerland

**Keywords:** Genetic architecture, multivariate statistics, pleiotropy, spectral decomposition, trait correlation

## Abstract

A key goal in evolutionary quantitative genetics is to understand how evolutionary trajectories are constrained by pleiotropic coupling among multiple traits. Because studying pleiotropic constraints directly at the molecular genetic level remains very difficult, several analytical approaches attempt to draw conclusions about constraints by relating the orientation of the eigenvectors of the traits' (co)variance matrix to vectors of multivariate selection. On the basis of explicit models of genetic architecture, I here argue that the value of such approaches is greatly overestimated. The reason is that eigenvector orientation can be highly unstable and lack a biologically meaningful relationship with the underlying traits' genetic architecture. Genetic constraints are more profitably explored through experimental approaches avoiding the mathematical abstraction inherent in eigenanalysis.

## Introduction

The major goal of evolutionary quantitative genetics (EQG) is to understand the rate and direction of evolution in multiple traits. A pivotal concept here is that traits generally cannot evolve independently because their genetic architecture is shared to some extent (Dickerson 1955; Lande and Arnold 1983; Cheverud 1984; Maynard Smith et al. 1985; Charlesworth 1990; Arnold 1992; Björklund 1996; Schluter 1996; Blows and Hoffmann 2005; Blows 2007; Agrawal and Stinchcombe 2009; Kirkpatrick 2009; Walsh and Blows 2009). That is, if a genetic locus influences multiple traits (pleiotropy), allele frequency shifts at this locus driven by selection on one trait will generate correlated responses in other traits. Genetic covariance among traits caused by pleiotropy can thus bias the rate and/or the direction of responses to selection relative to the situation where genetic variance in each trait is independent. Such bias arising from pleiotropy (or tight linkage between genetic factors) represents a form of *genetic constraint*. Identifying such constraints, and quantifying their strength, is the major avenue to understanding multivariate evolution in EQG (Blows 2007; Agrawal and Stinchcombe 2009; Kirkpatrick 2009; Walsh and Blows 2009).

**Figure 1:**
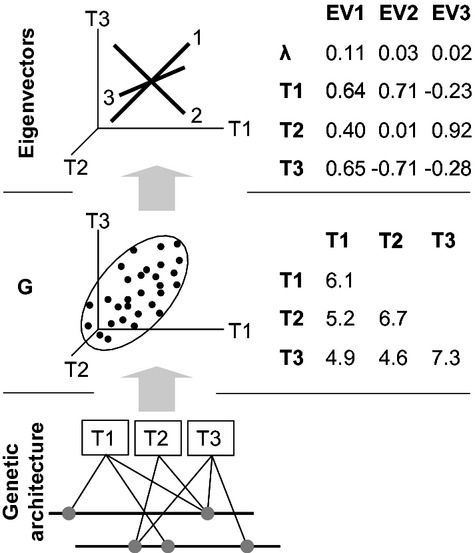
Schematic illustration of the two-step mathematical abstraction involved in eigenvector-based EQG studies of genetic constraints. The base level shows three traits (T1–T3) influenced by multiple genetic loci (gray dots on the thick black lines representing two different chromosomes). Some of the loci target more than one trait, hence act pleiotropically. This molecular genetic detail is very difficult to quantify directly, but can be summarized (first abstraction step; gray arrow) more easily in the additive genetic (co)variance matrix **G** (middle level). A graphical representation of **G** is given on the left. Here the dots indicate individual breeding values in T1–T3 space for the sampled population (hypothetical data), and **G** is visualized by the ellipse. **G** is given in matrix form on the right, with the diagonal elements being trait variances and off-diagonal elements being bivariate covariances. The second abstraction step involves the diagonalization of **G** to obtain its eigenvectors (top level; visualized on the left). The eigenvectors (EVs) are orthogonal axes (as many as underlying traits), each described by its eigenvalue and trait loadings (matrix on the right). Eigenvalues (λ) specify the amount of variation captured by each EV, hence define their magnitudes (maximal for EV1, minimal for EV3). Trait loadings (ranging from –1 to 1) specify the collinearity of an EV with the original trait axes, and hence define each EVs orientation in trait space.

Unfortunately, empirical information on how pleiotropy influences patterns of trait variance and covariance (hereafter simply [co]variance) is very difficult to obtain and highly incomplete even for model organisms (Maynard Smith et al. 1985; Barton and Turelli 1989; Roff 2007; Hill 2010), precluding the study of genetic constraints directly at the level of molecular genetic architecture. For this reason, a common approach taken in EQG investigations of genetic constraints involves a two-step abstraction away from molecular genetic architecture: first, trait-specific and pleiotropic aspects of genetic architecture are summarized across all genetic factors by estimating the additive genetic (co)variance matrix **G** (Fig. 1). Second, **G** is subjected to diagonalization (spectral decomposition) to obtain its eigenvectors (EVs). The EVs are described by their eigenvalue (quantifying magnitude) and trait loadings (quantifying orientation) and provide a representation of the total variation in **G** along orthogonal, variance-maximizing multivariate axes (details given in Fig. 1).

A fundamental but generally tacit assumption in EQG is that the orientation of **G**'s EVs still retains a meaningful connection to the molecular genetic architecture of the underlying traits. This would permit the interpretation of key aspects of genetic architecture based on the loadings of the constituent traits on their EVs, as these loadings define the orientation of EVs in trait space (Fig. 1). For example, “The direction of greatest genetic variance (**g**_**max**_) showed that genotypes at one extreme of the population had relatively short, slender bodies; narrow mouths; and numerous, long gill rakers” (Schluter 1996, p. 1769). (Note that **g**_**max**_ is the first EV of **G**.) A direct and interpretable connection between trait loadings on an EV, genetic (co)variance among traits, and underlying genotypes is here assumed (for similar examples see Lande and Arnold 1983, p. 1222; Renaud et al. 2006, p. 1707).

Based on the general assumption in EQG of a tight link between genetic architecture and the orientation of EVs, a family of techniques attempt to infer genetic constraints by relating the orientation of EVs to measured (or inferred) axes of selection. These approaches are hereafter called *EV-based approaches* to exploring genetic constraints. The best known of these methods is arguably Schluter's (1996) test for “evolution along lines of least genetic resistance” (related methods are reviewed in Walsh and Blows 2009). Here, the orientation of the first EV of **G** (estimated from a population assumed to represent the ancestral state) is compared with the orientation of the vector of multivariate selection (estimated from observed evolutionary trajectories among populations). A close directional association between the two vectors is taken as evidence that multivariate evolution has been biased by genetic constraints, although alternative interpretations are possible (Schluter 1996; Berner et al. 2010). The application of this and related EV-based analytical approaches has become increasingly popular (some recent examples: Blows et al. 2004; McGuigan et al. 2005; Renaud et al. 2006; Revell et al. 2007; Berner et al. 2008, 2010; Eroukhmanoff and Svensson 2008; Simonsen and Stinchcombe 2010; Colautti and Barrett 2011; Leinonen et al. 2011; Kimmel et al. 2012).

The fundamental assumption of a meaningful connection between the orientation of **G**'s EVs and genetic architecture, however, has been challenged. For instance, in response to an influential paper championing the use of matrix diagonalization in EQG (Blows 2007), Cheverud (2007, p. 15) argues that there “is nothing in the mathematical operation of spectral decomposition […] that has any necessary relationship with the biology underlying the traits” (see also Mitchell-Olds and Rutledge 1986; Houle et al. 2002; Brodie and McGlothlin 2007; Hunt et al. 2007). Surprisingly, such skepticism has had very little impact in EQG, perhaps because it has been based primarily on verbal argument rather than on evidence from formal analysis (but see Houle et al. 2002). The goal of the present study is therefore to demonstrate more directly that a biologically meaningful link between EV orientation and genetic architecture, and the corollary that the former can be used to explore genetic constraints, suffers potentially severe flaws. I will do so by comparing EVs with their underlying, explicitly modeled genetic architecture and the associated **G** matrix.

## Methods

The validity of the assumption that the orientation of **G**'s EVs is useful for investigating genetic constraints depends on two conditions. First, for a biological system with a specific genetic architecture, the orientation of the EVs (or at least the EVs of interest) should be relatively stable. If this criterion is not satisfied (i.e., if the orientation of EVs is highly contingent on the specific sample at hand), comparing the orientation of EVs to directions of selection will have little biological relevance. Second, trait loadings, which define an EV's orientation, should have a meaningful link to the traits' (co)variance and correlation structure, and to the underlying genetic architecture. Otherwise, interpreting the orientation of EV biologically (see quotes above) might be misleading, and forging a link between EVs and molecular genetic variation might be impossible. The analytical approaches described below are tailored to explore these two aspects, that is, the directional stability of EVs, and the relationship between EV loadings and genetic architecture.

### Directional stability of EVs

**Figure 2:**
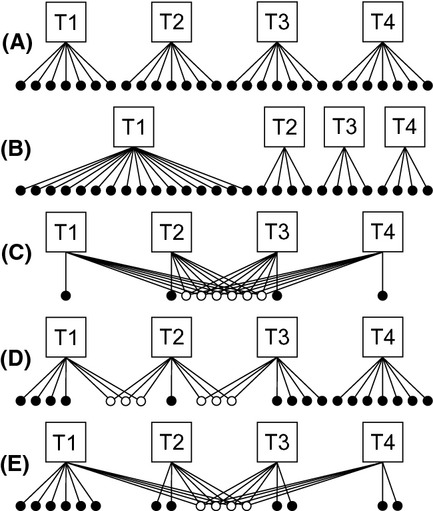
Genetic models used to explore the directional stability of **G**'s EVs (A–C), and the relationship between EV orientation (loading structure) and genetic architecture (D, E). Dots represent genetic loci determining the traits T1–T4 independently (black), or determining multiple traits simultaneously (pleiotropy; white). (A) Shows the default model for both the asymmetry and the pleiotropy series, that is, genetically independent traits with similar levels of variance. (B) Represents the most extreme level of asymmetry among traits in the magnitude of variance, while (C) visualizes the endpoint of the series modeling increasingly strong pleiotropic coupling among the traits. In (D), two traits (T1 and T3) are each independently coupled with T2. (E) is characterized by strong pleiotropy affecting all traits, and additionally by asymmetry among traits in the magnitude of variance.

The stability of the direction of **G**'s EVs in relation to different genetic architectures and to sample size was explored through a series of simulations. The default simulation approach involved the generation of a multivariate data set consisting of 100 individuals with four quantitative traits. Individual values for each trait were obtained by summing allelic states (–1, 0, 1; drawn at random with equal probability of 1/3) across seven independent genetic loci (Fig. 2A). This protocol generated exact breeding values for a set of four approximately normally distributed polygenic traits. In the default scenario, all traits displayed equal expected levels of variance, and their expected covariance was zero, producing an approximately spherical **G** matrix.

This default genetic architecture was then modified to model situations with increasingly strong asymmetry among the traits in their magnitude of variance (hereafter called the “asymmetry series”). This was achieved by raising the number of loci determining the first trait (T1) to 10, 13, and 16, while reducing the number of loci driving T2–T4 to 6, 5, and 4 (the endpoint of this series is visualized in Figure 2B). The total variance across the four levels in the asymmetry series thus remained constant.

In a second series (hereafter the “pleiotropy series”), I modified the default genetic architecture such that an increasing number of loci (1, 3, 6) were shared among all four traits. The strength of pleiotropic coupling among the traits thus increased gradually in this series, while the total variance again remained constant. The endpoint of this series, shown in Figure 2C, resembles a situation where a collection of morphological variables scales strongly with overall body size due to pleiotropic growth factors.

To examine the effect of sample size on EV stability, several genetic architectures from the asymmetry and pleiotropy series were modeled with sample sizes of 50, 200, and 400 in addition to the default sample size (100). The lowest sample size modeled seems to be representative of typical empirical studies: median sample size (i.e., the number of individuals in phenotypic studies; the number of full sib families or sires in genetic studies) across 18 haphazardly chosen EV-based studies of genetic constraints was exactly 50 (mean 66; maximum 196).

For each level of the asymmetry and pleiotropy series, and for the different sample sizes, data generation was performed in 1000 replicates. Each of the resulting data sets was used to compute the matrix of additive genetic (co)variances **G**, which was subjected to singular value decomposition to obtain the EVs. Directional stability of EVs in relation to variance asymmetry, the strength of pleiotropy, and sample size was then explored by evaluating trait loadings on the EVs. Note that for these simulation series, the presentation of results is limited to EV1, as this leading axis of trait (co)variance is usually considered the most important (e.g., Schluter 1996), and because interpreting the other EVs did not produce qualitatively different insights.

### Relationship between EV loadings and genetic architecture

Issues in the relationship between the direction of EVs and their underlying genetics are illustrated by two examples. In the first, I specified a genetic architecture involving two genetically independent traits (T1, T3), each of which was linked to T2 by three pleiotropic loci (Fig. 2D). T4 was independent from the other traits. The second example involved four pleiotropic loci influencing all traits. T1 was additionally determined by six independent loci, each of the three other traits by only two independent loci (Fig. 2E). This scenario thus involved both strong pleiotropy and asymmetry among the traits in their magnitude of variance.

To explore the connection between EV trait loadings and genetic architecture, I calculated the theoretical **G** matrix for each of the two scenarios based on the exact parametric (co)variance contributed by a single locus with the allelic states given above. (The exact value was 2/3, as determined empirically by using simulations with very high sample size.) This matrix was subjected to singular value decomposition to obtain the EVs. I then assessed qualitatively the relationship between trait loadings on the EVs and the trait's theoretical correlation structure derived from **G**. In addition, I plugged each **G**, along with a linear selection gradient vector ***β*** of 0.5, 0, 0, 0 (selection on T1 only), into the multivariate breeder equation 
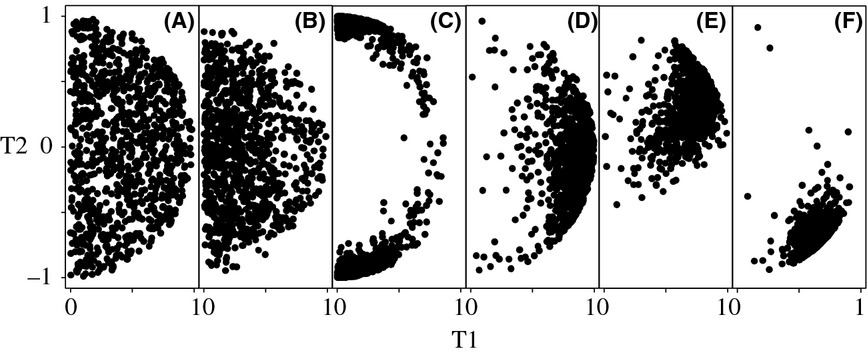
 (Lande 1979; 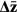 is the vector of changes in trait means over one generation of selection). Comparing 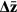 to the trait loadings on the EVs provided an alternative way to assess whether patterns of trait correlation indicated by the orientation of EVs had a biologically meaningful link to trait associations revealed by correlated responses to selection.

### Exploring the robustness of the analytical approaches

The robustness of the findings emerging from the above analyses was scrutinized in several ways. First, I modified the distribution of allelic values of the genetic loci. For instance, I here considered values drawn at random from a normal distribution, or allowed different loci to display different allelic ranges, thereby mimicking quantitative trait loci (QTL) with different effect sizes. Second, I doubled the number of both the independent and pleiotropic loci in each scenario. These modifications had no effect on the outcome of the simulations. I therefore limit the presentation of results to the allelic values and number of loci described above.

Second, I modified trait space dimensionality. The 18 EV-based studies mentioned above displayed a median trait number of nine. I therefore approximated this number by doubling the number of traits in all scenarios from four to eight (e.g., by adding four variables similar to T2–T4 in the asymmetry and pleiotropy series). These alternative analyses did not produce qualitatively novel results and are therefore not presented.

Data generation, analysis, and plotting were performed by using the R language (R Development Core Team 2010). Coding is provided on request.

## Results

### Directional stability of EVs

**Figure 3:**
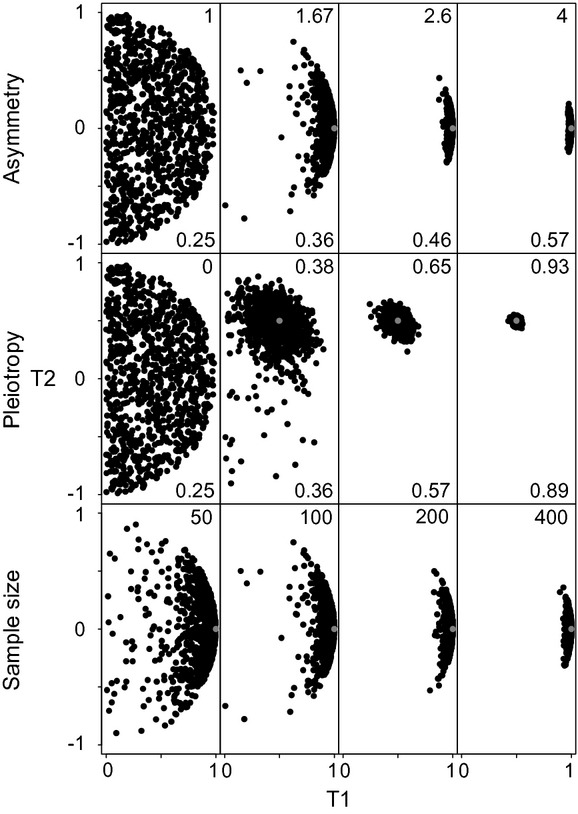
Loadings of the first and second trait (T1, T2) on the first eigenvector (EV1) across 1000 replicate simulations for three simulation series. Parametric loadings are superimposed as gray dots. Top row: gradual increase in the variance of T1 relative to the other traits (Asymmetry). Values within each panel indicate the parametric magnitude of variance in T1 relative to each of the other traits (top), and the parametric proportion of variance captured by EV1 (bottom). Middle row: increase in the number of loci determining all traits simultaneously (Pleiotropy). Values within each panel give the parametric correlation among the traits (top), and the parametric proportion of variance captured by EV1 (bottom). Sample size in both the asymmetry and pleiotropy series is 100. Bottom row: example of a series with increasing sample size (given within each panel). This series is based on the genetic architecture used to model the second level of the asymmetry series (i.e., T1 displaying 1.67-fold greater variance than each of the other traits). Note that to remove redundancy, the arbitrary polarity of the eigenvectors was always corrected for by simultaneously multiplying the loadings of both traits by –1 whenever T1 exhibited a negative loading. This thus coerced T1 (but not T2) to loadings ranging between 0 and 1.

The asymmetry series showed that trait loadings on the first EV, and hence the orientation of this vector, were poorly defined when traits were genetically independent *and* displayed relatively similar levels of variance. The orientation stabilized as the asymmetry in variance increased (and the sphericity of **G** thus decreased) (Fig. 3, top). Similarly, increasing magnitudes of pleiotropic gene action (leading to stronger correlations among the traits) rendered the orientation of EV1 increasingly consistent (Fig. 3, middle). Finally, the stability of the orientation of EV1 was dependent on sample size (Fig. 3, bottom). For a typical sample size used in empirical work (*N* = 50), trait loadings generally proved relatively inconsistent across replicate simulations.

### Relationship between EV loadings and genetic architecture

Scenario D modeled two sets of pleiotropic loci, each influencing T2 and either T1 or T3. This genetic architecture was mirrored in the traits' theoretical covariance and correlation structure (Table 1, top). In particular, there was a substantial correlation within the trait pairs T1–T2 and T2–T3, whereas T1 and T3 were uncorrelated. In line with these patterns, selection on T1 produced a strong direct response in that trait and a correlated response in T2, but no responses in the other traits. By contrast, the orientation of **G**'s EVs was misleading when subject to standard biological interpretation. For example, EV1 displayed strong loadings by T1, T2, and T3. This pattern would generally be taken as evidence for the presence of pleiotropic genetic factors shared among *all three* traits. In reality, however, T1 and T3 were genetically independent, but simply associated with the same third variable. Similarly, the strong but opposed loadings of T1 and T3 on EV2 might suggest the presence of an additional layer of antagonistically pleiotropic loci influencing this trait pair. Such loci, however, were not modeled.

**Table 1:** Covariance and correlation structure (Cor, Cov; correlations in boldface), response to selection, and eigenstructure (loadings of the traits T1–T4 on the eigenvectors EV1–EV4; proportion of total variance given in parentheses) for the scenarios D and E visualized in Figure 2. The multivariate response to selection (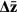) is over one generation with a multivariate linear selection gradient *β* of 0.5, 0, 0, 0 (i.e., selection on T1 only)

Scenario	Cor, Cov					Response	Eigenstructure			
D		T1	T2	T3	T4	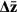	EV1 (0.40)	EV2 (0.25)	EV3 (0.25)	EV4 (0.1)
	T1	4.67	2.00	0.00	0.00	2.33	0.50	0.71	0.00	-0.50
	T2	**0.43**	4.67	2.00	0.00	1.00	0.71	0.00	0.00	0.71
	T3	**0.00**	**0.43**	4.67	0.00	0.00	0.50	–0.71	0.00	–0.50
	T4	**0.00**	**0.00**	**0.00**	4.67	0.00	0.00	0.00	1.00	0.00
E		T1	T2	T3	T4	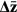	EV1 (0.69)	EV2 (0.17)	EV3 (0.07)	EV4 (0.07)
	T1	6.67	2.67	2.67	2.67	3.33	0.60	0.80	0.00	0.00
	T2	**0.52**	4.00	2.67	2.67	1.33	0.46	–0.35	0.00	0.82
	T3	**0.52**	**0.67**	4.00	2.67	1.33	0.46	–0.35	–0.71	–0.41
	T4	**0.52**	**0.67**	**0.67**	4.00	1.33	0.46	–0.35	0.71	–0.41

In scenario E, I specified a situation with both strong global pleiotropy and heterogeneity in the magnitude of variance among the traits. As expected, this produced strong correlations among all traits, and selection on T1 caused correlated evolution in all the other traits (Table 1, bottom). Intuitively consistent with these patterns, all traits loaded substantially on EV1, and this axis captured a high proportion of the total variance. When studying morphological traits, EV1 would here typically be considered a latent size variable determined by pleiotropy among all traits, and the remaining axes interpreted as size-independent shape axes (e.g., Lande and Arnold 1983; Schluter 1996; Merilä and Björklund 1999). The substantial loadings of opposed sign on EV2 between T1 and the other traits would thus be taken as evidence for strong antagonistic pleiotropy between T1 and the other traits, revealed after partialing out global size-related trait correlation. This latter biological interpretation is clearly misleading, as antagonistic pleiotropy was not modeled.

I emphasize that the results obtained from the scenarios D and E are not caused by symmetries built into the genetic architecture; similar findings emerged when giving each trait a unique magnitude of variance. Also, extending these scenarios to include more traits produced qualitatively similar results (details not presented).

## Discussion

Vector-based approaches in EQG rely on the assumption that the diagonalization of **G** yields axes that can be used to infer how genetic (co)variation due to pleiotropic gene action constrains trajectories of multivariate evolution. This assumption was here investigated, leading to two main insights.

First, the orientation of EVs associated with a given genetic architecture can be highly unstable. Perhaps not unexpectedly, one important contributor to EV instability is low sample size. Indeed, the simulations suggest that the typical sample sizes used in empirical work produce estimates of **G** imprecise enough to generate substantial fluctuation in the orientation of EVs under different genetic architectures. While estimation error has long been recognized as a general concern in EQG (Barton and Turelli 1989; Lynch and Walsh 1998; Pigliucci 2006), it is generally ignored in EV-based empirical work (for an exception see Leinonen et al. 2011).

But even with high sample sizes allowing a precise estimate of the genetic (co)variance structure, the directionality of **G**'s EVs will be unstable if the proportion of total variance (or the residual variance not accounted for by higher-level EVs) is distributed relatively evenly among the (remaining) EVs. This will be the case whenever traits exhibit relatively similar magnitudes of variance, and when pleiotropy is low (Fig. 3, top and middle). In other words, irrespective of sample size, it appears that the orientation of EVs is most stable in situations where EV-based multivariate methodology is least useful. That is, when each EV is driven disproportionally strongly by a single trait (for a striking empirical example see Berner et al. 2010), or by collections of traits that represent essentially redundant manifestations of the same genetic factors. In both situations, one could argue that responses to selection might be understood or predicted reasonably well within a simple univariate framework.

Instability of EV orientation suggests that conclusions about genetic constraints drawn from a directional comparison of EVs with multivariate axes of adaptive divergence or measured selection vectors can be highly contingent on the available sample; qualitatively different conclusions might be drawn when using a different sample from the same biological population. Note that this issue is not necessarily resolved by analytical approaches considering variability of EV orientation through resampling (e.g., Schluter 1996; Berner 2009). The reason is that when sample size is low, the orientation of an EV can be relatively stable across *resamples* of the same sample due to chance, even when EV orientation is completely unstable across *independent* replicate samples from the same statistical population (illustrated in Appendix 1).

The second major finding is that even when EVs are directionally stable, it cannot be taken for granted that their orientation has a meaningful relationship with the genetic architecture of the constituent traits, with the estimated (co)variance and correlation structure of the traits, and with the traits' responses to selection predicted by the multivariate breeder framework. The problem is that the diagonalization of **G** by definition produces mutually orthogonal vectors. Orthogonality, however, is simply not a property of molecular genetic architecture (Mitchell-Olds and Rutledge 1986; Brodie and McGlothlin 2007; Cheverud 2007; Hunt et al. 2007; see Berner 2011 for an analogous demonstration in a morphometric context). Treating the EVs of **G** as *genetically* (as opposed to mathematically) independent trait combinations is therefore flawed, and interpreting EV loadings in genetic or even functional terms is poor practice. Conclusions regarding genetic constraints on multivariate evolution drawn in EV-based EQG studies should be taken with skepticism.

I emphasize that the present investigation does not demonstrate that the identified analytical difficulties will necessarily, or equally strongly, compromise *any* EV-based analysis of genetic constraints. The problem is, however, that with complex real-world data, even the evaluation of eigenvalues and/or the inspection of the genetic correlation structure among traits might provide little guidance as to whether the assumption of a meaningful association between any EV and genetic architecture is justified.

The findings presented in this study also question the value of EVs as a bridge between phenotype and genotype maps (Houle 2010), or as a tool for summarizing pleiotropic gene action in multivariate selection analysis (Lande and Arnold 1983; McGuigan et al. 2011). Subjecting collections of traits to eigenanalysis certainly allows us to identify multivariate composite axes of variation, and tell us how total variation is distributed among these axes (Mezey and Houle 2005; Hine and Blows 2006; Blows 2007; Walsh and Blows 2009). But we should not expect that this exercise will inform on how phenotypes are related to genotypes, or illuminate the link between fitness, traits, and their genetic architecture (Mitchell-Olds and Rutledge 1986; Mitchell-Olds and Shaw 1987). An instructive illustration of the disconnect between EV orientation and genetic architecture is provided by a mapping study searching for QTL underlying body shape differentiation between two stickleback fish populations (Albert et al. 2008). This study failed to find QTL when analyzing the principal components (PCs) of shape variables (note that PCs are obtained by projecting trait values on the EVs, hence PCs and EVs have identical orientation). The analysis of the raw shape variables (avoiding matrix diagonalization), however, identified many shape QTL, including large effect loci. This highlights that EVs are mathematical constructs whose orientation may not have a traceable relationship with genetics.

If approaches relying on the orientation of EVs are inappropriate to explore genetic constraints, what alternatives are available? Several EQG methods have been introduced that make use of the summary statistic **G**, but that avoid the additional mathematical abstraction inherent in the diagonalization of this matrix. These methods include comparing the magnitude of variance in the direction of evolution to the magnitude of variance in random directions (Hansen and Houle 2008), or comparing the rate of adaptation given an observed **G** matrix to the rate of adaptation predicted if all traits were genetically independent (Agrawal and Stinchcombe 2009). Such approaches relying on **G**, however, also face a number of potentially serious problems (reviewed in Mitchell-Olds and Rutledge 1986; Barton and Turelli 1989; Pigliucci 2006). These include parameter estimation difficulties mentioned earlier, the instability of the (co)variance structure, the influence of unmeasured traits, nonadditive genetic effects, and that identical **G** matrices can emerge from very different genetic architectures.

To summarize, it may be debatable whether observational EQG approaches, in general, are the most effective route to understanding multivariate evolution, or whether efforts are more profitably invested in manipulative experimental approaches, such as artificial selection and the measurement of correlated responses (Mitchell-Olds and Shaw 1987; Barton and Turelli 1989; Fry 1993; Brakefield and Roskam 2006; Roff 2007; for examples see Palmer and Dingle 1986; Bradshaw and Holzapfel 1996; Mitchell-Olds 1996; Beldade et al. 2002; Conner et al. 2011). Certainly, however, we are deluding ourselves if we expect much progress in understanding multivariate evolution from the application of methods relying on the directionality of **G**'s eigenvectors.

## Appendix 1

Directional instability of EVs, as revealed by trait loadings across multiple *replicate* samples for a given genetic architecture, might not be apparent when examining trait loadings across bootstrap *resamples* of a specific sample. This is here illustrated based on the genetic architecture involving four traits with equal expected variance and no pleiotropy (the default genetic architecture used for the simulations; see Methods for details). For this specific genetic architecture, **G** is spherical and hence the orientation of EVs is undefined, as reflected in the unstable loadings of T1 and T2 on EV1 across 1000 replicate samples (panel A; identical to the left panel of the “Asymmetry” series in Figure 3). The EV instability seen among replicate samples, however, may or may not be observed across bootstrap resamples (*N* = 1000) of the replicate samples (panels B–F show selected examples). In B, for instance, the bootstrap trait loadings mirror high instability of EV1, whereas the consistent bootstrap loading structure in F reflects a highly stable orientation of EV1. The latter is due to a biologically trivial deviation of **G** from sphericity, causing EV1 to capture a slightly greater proportion of total variance (e.g., 0.36 in the sample underlying F as opposed to 0.3 in the sample underlying B). Qualitatively similar effects were observed when modeling nonspherical **G** matrices. Note that the potential for bootstrap resampling to underestimate the EV instability inherent in a given genetic architecture increases as sample size decreases (and hence the leverage of each specific data point increases; details not presented).
